# Computational Design of a DNA- and Fc-Binding Fusion Protein

**DOI:** 10.1155/2011/457578

**Published:** 2011-09-14

**Authors:** Jonas Winkler, Giuliano Armano, J. Nikolaj Dybowski, Oliver Kuhn, Filippo Ledda, Dominik Heider

**Affiliations:** ^1^Department of Bioinformatics, Center for Medical Biotechnology, University of Duisburg-Essen, Universitaetsstra*β*e 1-5, 45117 Essen, Germany; ^2^Department of Electrical and Electronic Engineering, University of Cagliari, Piazza d'Armi, 09123 Cagliari, Italy

## Abstract

Computational design of novel proteins with well-defined functions is an ongoing topic in computational biology. In this work, we generated and optimized a new synthetic fusion protein using an evolutionary approach. The optimization was guided by directed evolution based on hydrophobicity scores, molecular weight, and secondary structure predictions. Several methods were used to refine the models built from the resulting sequences. We have successfully combined two unrelated naturally occurring binding sites, the immunoglobin Fc-binding site of the Z domain and the DNA-binding motif of MyoD bHLH, into a novel stable protein.

## 1. Introduction

Protein design methods use trial and error or more sophisticated methods like directed evolution or inverse folding to generate novel scaffolds or to find novel protein sequences folding into a defined scaffold, respectively. Given the intimate relationship between a protein's structure and function, a way to design proteins with targeted properties is to start from a desired structure and find sequences able to fold into it, imposing additional constraints in the process [1]. On the one hand, it is known that, in general, similar sequences fold into similar structures [2]; on the other hand, there are many cases of nearly identical structures known, sharing no sequence similarity at all [3]. However, the aim of computational design methods is not finding all possible solutions, but at least one solution that fits the required properties. One of the methods that have been proposed is a multiobjective optimization, in which protein stability and catalytic activity are simultaneously optimized [4, 5].

In convergent evolution, nonhomologous proteins evolve in separate biological contexts to catalyze the same or similar reactions. There exist two types of convergent evolution: (1) mechanistic analogs that uses the same mechanisms to perform related reactions and (2) transformational analogs catalyzing exactly the same reaction. However, analogous proteins may have structural homology although this is not a prerequisite. Prominent examples are the antifreeze glycoproteins [6], protein phosphatases [7], and glutaminyl cyclases [8].

Several methods have been proposed to design novel stable proteins, such as multi-objective optimization, in which protein stability and catalytic activity are simultaneously optimized. For instance, Gronwald et al. [4] used a multi-objective optimization to build new stable peptides based on the villin headpiece (VH) sequence, which is known to be stable *in vitro*. VH is derived from a single protein domain of 35 residues [9]. The algorithm of Gronwald et al. consists of four steps. First, the sequences carrying point mutations are modeled on a given template structure, and subsequently, molecular dynamics simulations are carried out for 10 ns. After simulation, the fitness of each model is evaluated, and the best models are selected for further optimization.

The limits of current methods is the incorporation of molecular dynamics simulations into the multi-objective optimization. Due to the fact that molecular dynamics simulations are very expensive regarding computational time, new fitness functions have to be introduced without loosing predictive power. Thus, a preprocessing and prescreening of amino acid sequences is necessary due to the huge dimension of the potential sequence space. In classification studies, amino acids are often represented by so called descriptors, mapping each amino acid to a numerical value. These descriptors range from physicochemical properties, for example, hydrophobicity, molecular weight, or isoelectric point, to more complex arrangements. It has been shown that the composition of the descriptor set is one of the most crucial parts in classifier development [10]. However, we tested several of these descriptors in different classification studies, ranging from functional classification and identification of protein families [11, 12], coreceptor prediction of HIV-1 [13], and HIV-1 drug resistance prediction [14]. Hydrophobicity was one of the most important physicochemical properties, due to the fact that it is involved in protein interactions, for example, by forming hydrophobic cores. However, molecular weight is also important due to potential steric incompatibilities within protein cores. Furthermore, we found out that electrostatic potentials are also good descriptors, because they are also involved in protein interactions [13].

While most protein design methods focus on divergent evolution, and thus aim at improving characteristics of a specific protein such as stability and binding affinity, we used directed evolution to create a novel synthetic protein combining two unrelated naturally occurring binding sites: the immunoglobin Fc-binding site of the Z domain and the DNA-binding motif of MyoD bHLH. The resulting protein should be able to bind to both the Fc region of human antibodies and to DNA simultaneously. We compare our multiobjective optimization scheme to that of Gronwald et al. [4] with respect to computational efficiency and overall number of sequences investigated.

## 2. Materials and Methods

### 2.1. Protein Z and MyoD

Protein Z is derived from staphylococcal protein A and holds an IgG Fc-binding domain. It consists of a three-helix bundle built from 58 amino acids. Helix 1 and 2 contain the Fc-binding region, whereas helix 3 is necessary for Fab binding [15]. Chain B of PDB file 1LP1 [16] was used as a model for protein Z. In this study, we transplanted the DNA-binding region of MyoD intro helix 3 of protein Z. MyoD is a bHLH domain DNA-binding protein [17]. The protein-DNA complex structure (PDB: 1MDY) was used in this study.

### 2.2. Design Process

**Figure 1:**
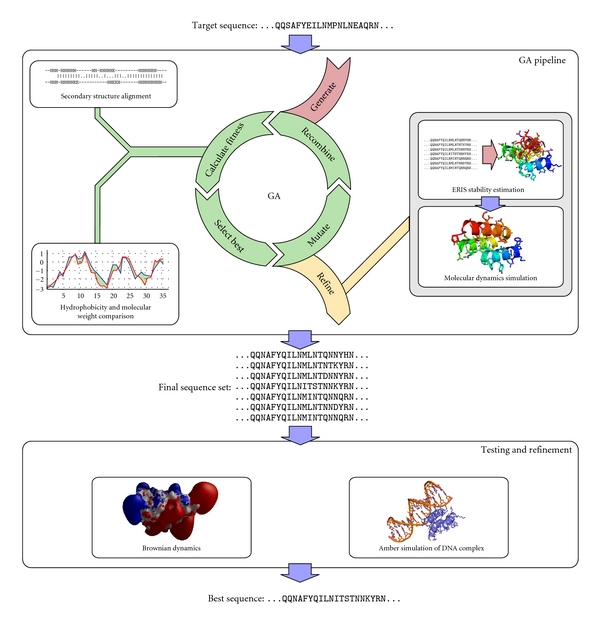
Chart of the design process. We employed a genetic algorithm (GA) with a fitness function based on secondary structure alignments and hydrophobicity and molecular weight comparisons. The resulting sequence set of this iterative process was refined using ERIS to build and rank the models which were then simulated using molecular dynamics simulations in order to estimate stability according to [4]. Amber and Brownian dynamics simulations are applied for testing and refinement of the final optimized protein models.

We employed a genetic algorithm (GA) with a multiobjective fitness function based on secondary structure alignments and hydrophobicity and molecular weight comparisons. In an iterative process, sequences were assessed by the fitness function, best-ranked sequences were selected, recombined, and mutated to get new sets of sequences. The resulting sequence sets were refined in a second step. ERIS [18] was used to model sequences onto the wild-type structure and to calculate their free energy. The models with the lowest free energy were subsequently evaluated using molecular dynamics simulations (Figure 1).

### 2.3. Multiobjective Optimization

Multiobjective optimization has been widely applied in protein design [4], providing a heuristic solution for optimization problems without the need for problem specific domain knowledge.

The quality of a solution is not represented by a single value, but rather as a vector representing the quality for each criterion. This can be formulated as

(1) $$\begin{array}{c} \left\{f_{1}\left(x\right),f_{2}\left(x\right),\ldots ,f_{n}\left(x\right)\right\}\in {\mathbb{R}}^{n}, \end{array}$$

with *f*_*i*_ being the corresponding fitness functions and *x* the target protein.

In contrast to natural or real numbers, vectors do not have a natural order. To compare vectors with each other, which is necessary for the optimization process, we identify all vectors dominated by another one. One vector dominates another vector if it is bigger in at least one component and equal at the remaining components. This can be mathematically expressed by *x* and *y* being the vectors to be compared:

- *x* = *y*⇆*x*_*i*_ = *y*_*i*_,   for  all  *i* = 1,…*n*,
- *x* > *y* → ∃*i* ∈ 1,…, *n* with *x*_*i*_ > *y*_*i*_ and *x*_*j*_ ≥ *y*_*j*_, for  all  *i* ≠ *j*,
- if *x* has greater and smaller components than *y*, the vectors do not dominate each other.

For instance, *x* = (3,1, 2) dominates *y* = (3,1, 1), because *x*_3_ > *y*_3_. However, *z* = (4,0, 2) neither dominates *x* nor *y*, because *z*_2_ < *y*_2_ = *x*_2_.

All vectors that are not dominated by other vectors build the first *Pareto frontier*. Dominating vectors are removed from the set, and the next *Pareto frontiers* are calculated iteratively, thus leading to a Pareto rank count. Vectors having a lower Pareto rank count are more likely selected for a new generation. Two individuals are chosen and combined using 1-point-crossover at a random position leading to a new individual. This novel individual is subsequently mutated at a random position. All individuals, including those from the current generation and the newly generated ones, are ranked based on their fitness, and the best individuals are selected for further evolutionary optimization, whereas the worst individuals are discarded. Thus, the number of individuals per generation is fixed (here: 600 individuals).

### 2.4. Scoring Functions

Secondary structure predictions were carried out with GAMESSP, a secondary structure predictor based on the GAME-framework [19]. GAMESSP is a multiple-expert-based secondary structure prediction software based on the PSIPRED algorithm [20], where each expert represents an independent artificial neural network. We used a basic secondary structure alphabet, namely, alpha-helix, beta-sheet and loop. GAMESSP was modified to use a local version of the SwissProt Database [21] due to performance purposes. As GAMESSP is written in Java, it can be easily adapted. The secondary structure predictions of the query and the target protein were aligned using a local alignment algorithm [22] to achieve a fitness score.

Hydrophobicity predictions were based on a sliding window procedure with a window size of seven [23]. The generated protein sequences were then ranked by the difference of the hydrophobicity integrals

(2) $$\begin{array}{c} \left|\overset{n}{\underset{0}{\int }}f_{\text{query}}\left(x\right)dx-\overset{n}{\underset{0}{\int }}f_{\text{target}}\left(x\right)dx\right|, \end{array}$$

with function *f* defined by the hydrophobicity values of the amino acids as splines and *n* being the length of the sequence. We used the hydrophobicity integrals instead of the single discrete values for the amino acid sequences to capture neutralizing effects of neighboring amino acids. In the same manner, the molecular weight scores were calculated using the molecular weights of the amino acids. 

### 2.5. Modeling New Sequences

The sequences from the first *Pareto frontier* were modeled on the query structure using ERIS [18]. ERIS was developed to handle more than one mutation with no loss of accuracy to predict protein stability. Protein Z (Chain B of PDB file 1LP1 [16]) was used as a template. ERIS performs free energy calculations by using prerelaxation of a template. Models of each sequence were built using flexible backbones.

### 2.6. MD Simulations of the Protein Models

Simulations of the protein models (build with ERIS) were performed using Gromacs 4.0.7 [24]. NVT ensembles were used for simulation of 20 ns. The leap-frog algorithm was used as an integrator with a 2 fs time step. Fast Particle-Mesh Ewald electrostatics (PME) were used with a 0.9 nm cutoff and the Van-der-Waals cutoff was set to 1.4 nm. Temperature coupling was set to Nose-Hoover, the reference temperature was set to 300 K. H-bonds were constrained using the linear constraint solver (LINCS). Protein stability was assessed by analyzing RMSD and RMSF.

### 2.7. MD Simulations of the Protein-DNA Complex

Simulations of the protein-DNA complexes were performed using Amber 10 [25]. Protein and DNA were described with the Amber99SB force field. Protons were added using the LEAP module. Each protein-DNA complex was immersed in an octahedral box of TIP3P water molecules that extended at least 10 Å outside the complex. Simulations were performed with the pmemd module in Amber 10. The SHAKE algorithm has been used to allow for an integration time step of 2 fs. Long-range interactions were treated with PME. The nonbonded cutoff was set to 9 Å. Langevin thermostat and Berendsen barostat were used. First, water molecules and hydrogens were minimized with 100-step steepest descent followed by 100-step conjugate gradient keeping all other atoms restrained with a force constant of 100 kcal/mol Å^2^. The solute was then minimized with 1000-step steepest descent followed by 1000-step conjugate gradient with no restraints. The system was gradually heated from 0 to 300 K over 10 ps in the NVT ensemble. 10 ns production simulation were carried out in the NPT ensemble.

### 2.8. Brownian Dynamics Simulations

Brownian dynamics simulations were carried out with BrownDye (http://browndye.ucsd.edu/) using the Northrup-Allison-McCammon method [26]. Protein-DNA reaction sites of the studied complexes were defined based on structural protein-DNA interactions described elsewhere [27]. Interacting atom pairs between molecules were defined as such if they formed a polar interaction at less than 4.5 Å distance. During diffusion simulations, successful association of molecules was assumed if three or more interacting atom pairs of diffusing and fixed molecules were closer than 5.5 Å. Each experiment consisted of 25.000 trajectories from which association rate constants were computed with BrownDye with a ionic strength of 0.3 mol/L.

## 3. Results and Discussion

**Figure 2:**
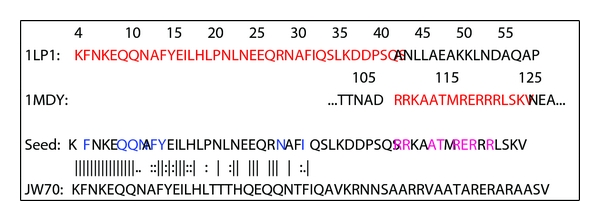
1LP1: sequence of the Z domain. 1MDY: part of the sequence of MyoD. Red marked amino acids are used as part of the seed sequence. Seed: seed sequence for the optimization. The blue and magenta marked amino acids are fixed during optimization. The initial population was created by randomly mutating black marked amino acids. JW70: selected model of the optimization aligned to the seed sequence.

We have successfully combined two independent binding sites into a given protein scaffold (PDB: 1LP1) using a genetic algorithm for sequence optimization. The fused sequence of the Z domain and MyoD was used as a start sequence (see Figure 2). Helix 3 of the Z domain (residue 42 to 57) was replaced by a DNA-binding helix of MyoD (residue 110 to 125). Amino acids essential for binding of Fc (5,9–11,13,14,28,31) and DNA (110,111,114,115,117–119,121) were conserved, while remaining positions were mutated during the optimization. The initial population consisted of randomly mutated seed sequences.

**Figure 3:**
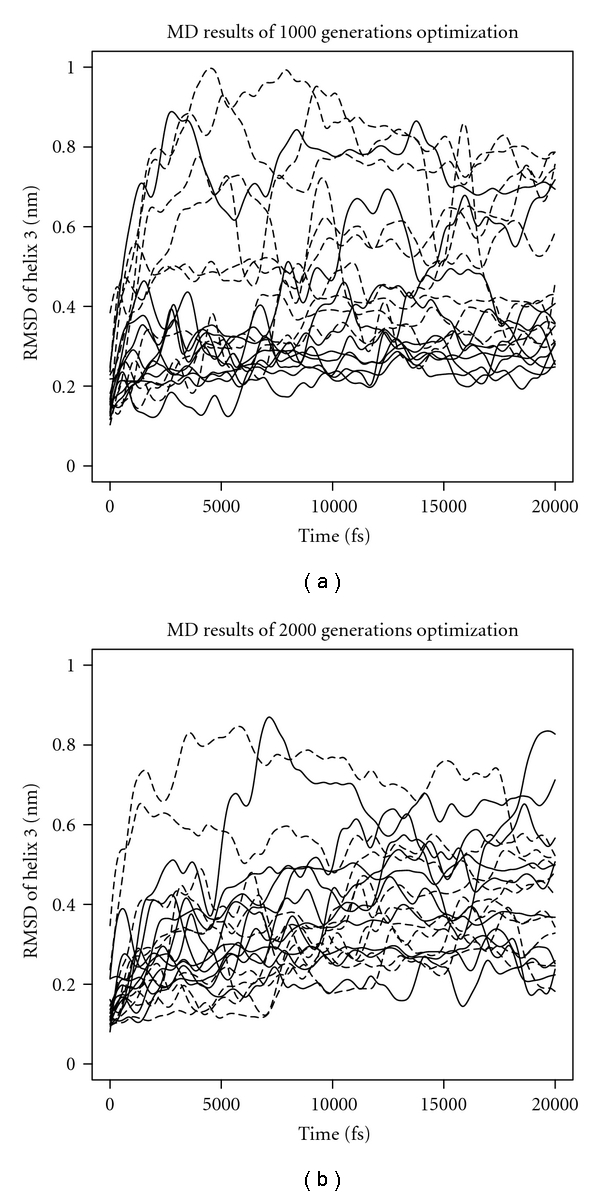
RMSD plots of the best (solid line) and worst (dashed line) sequences ranked by ERIS after 1000 generation (a) and 2000 generations (b), respectively. Models after 20 ns MD simulations were aligned to the wild-type structure of the Z domain using residues 6–17 and 22–33 (Helix 1 and 2). *C*_*α*_ RMSD of Helix 3 (residue 39–53) was calculated and smoothed using spline interpolation.

We simulated 1000 and 2000 generations, with each generation consisting of 600 individuals. A mutation rate of 0.01 led to a *Pareto frontier* of 67 and 86 individuals, respectively. Individuals were ranked using ERIS, and we carried out MD simulations of both the ten best ranked and the ten worst ranked individuals. After MD simulations, the models were aligned to the wild-type structure of the Z domain using residues 6–17 and 22–33 (Helix 1 and 2). *C*_*α*_ RMSD of Helix 3 (residue 39–53) was calculated and smoothed using spline interpolation (see Figure 3).

**Figure 4:**
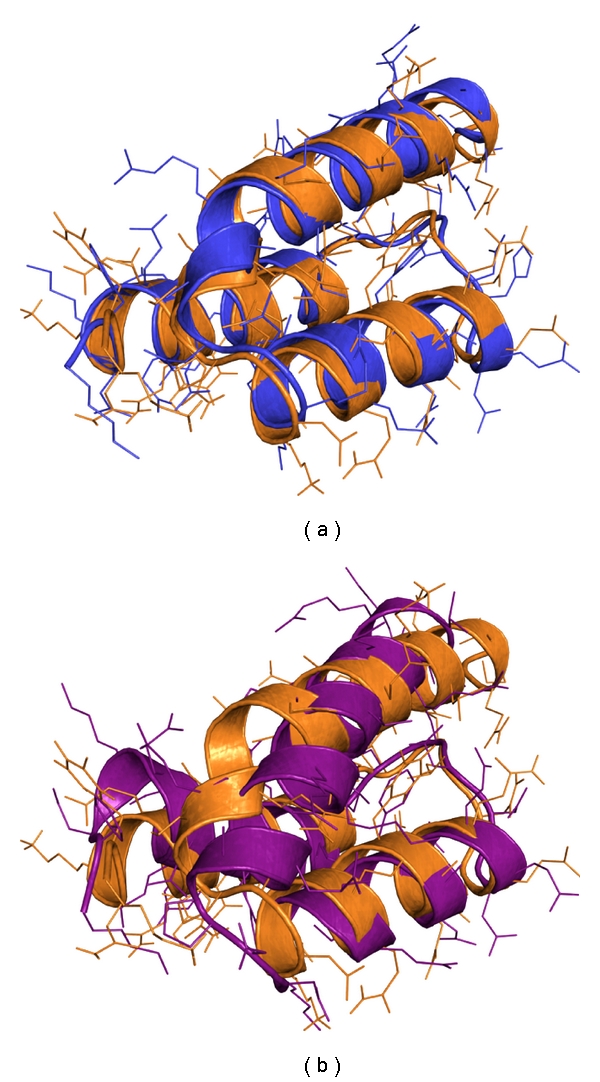
(a): JW70 after 20 ns MD simulation (blue) aligned to the structure of the Z domain from 1LP1 after 10 ns MD simulation (orange). (b): model of the seed sequence after 20 ns MD simulation (purple) aligned to the Z domain from 1LP1 (orange). Helix 3, which contains the new DNA-binding site, is shown on top.

 After 1000 generations of optimization, ERIS was able to separate low from high RMSD sequences (see Figure 3, left) very well. This is probably due to a widely spread *Pareto frontier*. After 2000 generations, optimization led to a narrow *Pareto frontier*, and thus ERIS was not able to distinguish the sequences anymore (see Figure 3, right). In addition, RMSF calculations of sequences optimized for 2000 generation showed improved stability of Helix 3 in comparison to the sequences that were optimized for 1000 generations. We then selected a model (JW70) with an all-atom RMSD of about 5 Å compared to the wild-type structure and an RMSD of about 1 Å to its starting structure, which implied a well-conserved geometry. Simulations of the seed sequence modeled on the 1LP1 structure as a negative control, showed dislocation of the three helices and thus potential negative effects to the functionality of the protein (see Figure 4).

As mentioned before, four Amber simulations were performed to check the models DNA-binding abilities. Protein-DNA interactions were modeled based on the 1MDY structure. We analyzed the interactions of DNA with our optimized fusion protein (JW70) as well as the interaction of DNA with the Z domain as a negative control, the seed sequence before optimization, and the MyoD-binding helix as a positive control. Both JW70 and the positive control bound stable to the DNA over 10 ns of simulation, while the negative control diffused from the DNA. The model of the seed sequence also bound to the DNA but lost its stability and partially unfolded.

In order to further assess the relative DNA-binding ability, we performed several Brownian dynamics (BDs) simulations to estimate the relative association rate constants (*k*_*on*_) of our models to the wild-type structure. As reference the wild-type protein-DNA complex (PDB: 1MDY) was used. The *k*_*on*_ of the reference WT complex was estimated to be 4.66·10^8^ M^−1^ s^−1^. The negative control protein, the native Z-Domain, did not associate with the DNA molecule in any of the 25.000 simulations, which is feasible considering its negative net charge and the absence of a DNA-binding site. All models generated during the optimization process, including the seed model achieved protein-DNA association, however, at varying estimated rates.  Table 1 summarizes the results. The most promising model JW70 showed a similar association rate relative to the WT (4.56·10^8^ M^−1^ s^−1^). The model seed, which was shown to retain DNA-interaction during a 10 ns MD simulation before partially unfolding, showed a reduced but still considerable *k*_*on*_ of around 25% relative of that estimated for the WT. All of these proteins have net charge of +5. In order to explore the effect of the net charge on the estimated *k*_*on*_, we included three more model into the analysis. JW19, JW56, and JW15 have net charges of +3, +5, and +7, respectively. Although there seems to be a logical trend of models with higher net charge associating with the target DNA more often, none of the other tested models achieved rates similar to JW70 and the WT.

In comparison to Gronwald et al. [4], we used a fusion protein of 56 residues instead of villin headpiece (36 residues). However, computational efficiency can be clearly compared (see Table 2). Gronwald et al. analyzed two runs of the multi-objective optimization with 15 generations, each consisting of 8 individuals. Thus, they carried out 240 MD simulations for 10 ns. The total CPU time was about of 1 year [4]. Our algorithm was able to analyze 600 individuals in 2000 generations, resulting in a total number of 1.2 million protein sequences. These huge number of sequences was analyzed in only 2 months, reflecting the high computational efficiency of our method compared to that of Gronwald et al.

## 4. Conclusion

We have applied multi-objective optimization guided by directed evolution to combine the MyoD DNA-binding motif into the Z domain conserving the scaffolds structure. Simulations showed that the optimization of the sequences based on hydrophobicity, molecular weight, and secondary structure predictions improved structural stability while maintaining protein functionality. The use of simple fitness functions reduces the optimization complexity, and thus allows to optimize more individuals over more generations resulting in a better sampling of the sequence space. 

## Figures and Tables

**Table 1:** Brownian dynamics simulation results.

model	*k*_*on*_(M^−1^ s^−1^)	net charge	rel. *k*_*on*_ (WT)
WT	4.66 · 10^8^	+5	1.000
Negative	0	−2	0.000
Seed	1.17 · 10^8^	+5	0.251
JW15	3.06 · 10^8^	+7	0.657
JW19	1.60 · 10^7^	+3	0.034
JW56	4.61 · 10^7^	+5	0.099
JW70	4.56 · 10^8^	+5	0.978

**Table 2:** Method comparison.

method	residues	individuals	generation	sequences	CPU time
GHH [4]	36	8	15	2 · 120	1 year
current study	54	600	2000	1.2 mil	2 months
